# Mycotoxin Biosensor Based on Optical Planar Waveguide

**DOI:** 10.3390/toxins10070272

**Published:** 2018-07-03

**Authors:** Ali Al-Jawdah, Alexei Nabok, Radhyah Jarrah, Alan Holloway, Anna Tsargorodska, Eszter Takacs, Andras Szekacs

**Affiliations:** 1 Materials & Engineering Research Institute, Sheffield Hallam University, Sheffield S1 1WB, UK; a.nabok@shu.ac.uk (A.N.); engafh@exchange.shu.ac.uk (A.H.); 2 Department of Physics, Faculty of Science, The University of Kufa, Najaf 54001, Iraq; rathyah@yahoo.com; 3 Department of Chemistry, The University of Sheffield, Sheffield S3 7HF, UK; a.tsargorodska@sheffield.ac.uk; 4 Agro-Environmental Research Institute, NARIC, Gödöllő 2100, Hungary; e.takacs@cfri.hu (E.T.); a.szekacs@cfri.hu (A.S.)

**Keywords:** optical biosensor, planar waveguide, polarization interferometer, refractive index sensitivity, mycotoxins

## Abstract

The research aim of this work is to develop a simple and highly sensitive optical biosensor for detection of mycotoxins. This sensor is built on a planar waveguide operating on the polarization interferometry principle, i.e., detecting a phase shift between p- and s-components of polarized light developed during the binding of analyte molecules. The operation of the proposed sensor is similar to that of a Mach–Zehnder interferometer, while its design is much simpler and it does not require splitting the waveguide into two arms. The refractive index sensitivity of the polarization interferometer sensor was in the range of 5200 radians per refractive index unit (RIU). Several tests were conducted to detect ochratoxin A (OTA) at different concentrations in direct immunoassay with specific antibodies immobilized in the sensing window. The lowest concentration of OTA of 0.01 ng/mL caused a phase shift of nearly one period. The results obtained prove high sensitivity of the sensors, which are capable of detecting even lower concentrations of mycotoxins at the ppt (part-per-trillion) level.

## 1. Introduction

At present, the detection of toxins is one of the main tasks for environmental science, security, agriculture, the food industry, and medicine. There is particular interest in detection of mycotoxins, products of the metabolism of numerous fungi species, which appear to have toxic, carcinogenic, and hormone-disruptive effects in humans [1]. Worldwide legislation sets quite strict limits on mycotoxin content in food and feed, typically at the ppb (part-per-billion) concentration level [2], which makes the detection of small mycotoxin molecules (with typical molecular weight in hundreds of daltons) a difficult task. Existing high-tech detection methods such as HPLC and mass spectroscopy can provide the required sensitivity, but such methods are expensive and time-consuming. Therefore, there is a great demand for development of biosensors for toxin detection. Highly sensitive optical immunosensors are leading in this development [3].

Our previous research exploiting the method of total internal reflection ellipsometry (TIRE) combined with direct immunoassay showed high sensitivity (in the sub-ppb range) for detection of different mycotoxins [4,5,6]. The use of a planar waveguide (PW) operating as a polarization interferometer (PI) [7] is a logical continuation of this work toward the development of portable sensor devices. The advantages of PW PI devices, due to their thousands of reflections of light, were demonstrated in [7,8], which may lead to the development of highly sensitive optical biosensors capable of label-free detection of toxins [3,6]. Several successful biosensors based on planar waveguides have been demonstrated recently. An inhibition sensor array based on PW optrodes using enzymes as bioreceptors was capable of detecting traces (in the sub-ppb concentration range) of heavy metals and pesticides in water [9]. The mainstream development of optical PW-based sensor devices lies in the use of Mach–Zahender (MZ) interferometers [10,11,12,13,14] and ring resonators [15,16], both approaches having demonstrating remarkable refractive index sensitivity around 7000 to 8000 rad/refractive index unit (RIU) [3] and versatility in their application. The development of a fully integrated all-silicon MZ biosensor [17,18,19,20] is particularly attractive and may lead to fabrication of portable, highly sensitive optical biosensors suitable for in-field or point-of-care detection of analytes of interest.

The main purpose of this work was to further explore the use of a much simpler PW sensor design (as compared to MZ-based devices) operating as a polarization interferometer with a view toward developing portable and highly sensitive devices capable of detecting low-molecular-weight molecules such as mycotoxins, particularly ochratoxin A, in very low concentrations in the ppt range.

## 2. Interferometric Sensor System

**Figure 1:**
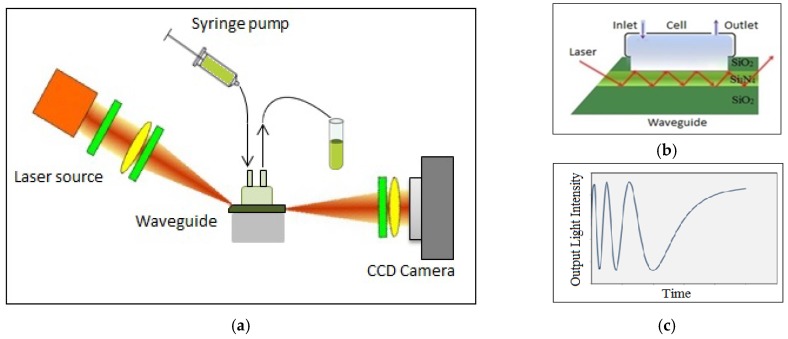
Designs of (**a**) planar waveguide and (**b**) polarization interferometer (PI) experimental setup; (**c**) expected output signal waveform.

The planar waveguide structures (shown in Figure 1a) were produced on silicon wafers using standard microelectronic processes and consisted of a thin (200 nm) layer of ${\mathrm{Si}}_{3}N_{4}$ sandwiched between much thicker (3 μm) layers of ${\mathrm{SiO}}_{2}$. Due to the large difference in the refractive indices of ${\mathrm{Si}}_{3}N_{4}$ core (n = 2) and ${\mathrm{SiO}}_{2}$ cladding (n = 1.46), the light propagates at an angle of 47° and thus experiences about 800 reflections per mm according to calculations based on the Goos–Hänchen effect [21].

In the experimental polarization interferometer (PI) setup in Figure 1b, a 635 nm light from a fan-beam laser diode was first made circularly polarized by λ/4 plate, then focused to a narrow strip using a semicylindrical lens and coupled to the waveguide through the slanted edge. At the other side of the waveguide, the light was collected with a charge-coupled device (CCD) array. A polarizer placed in front of the CCD camera allows the conversion of a phase shift between p- and s-components of polarized light into variations of light intensity (Figure 1c).

To monitor biochemical reactions, a window was etched in the top ${\mathrm{SiO}}_{2}$ layer, which brings the liquid sample in contact with the waveguide core. The reaction cell is sealed against the window and has inlet and outlet tubes to allow the injection of required liquids into the cell, and thus the adsorption of biomolecules on the surface of ${\mathrm{Si}}_{3}N_{4}$. Any changes in either the refractive index or the thickness of the adsorbed molecular layer affects mostly the p-component of polarized light, while the s-component acts as a reference, resulting in a multiperiodic output signal (Figure 1c):

$$V_{out}=V_{0}\cos(\Delta \phi )$$

where $\Delta \phi =\phi_{p}-\phi_{s}$ is the phase shift between the p- and s-components of polarized light.

**Figure 2:**
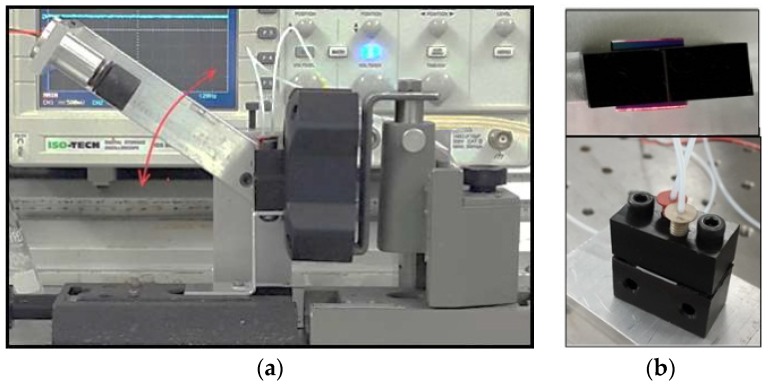
Photographs of (**a**) PI experimental setup and (**b**) reaction cell with inserted waveguide.

The photographs in Figure 2 show a general view of the PI biosensor setup (2a) and the cell with the inserted waveguide (2b) and the light coupling through the waveguide slanted edge. A Thorlabs (UK) LC100–Smart Line Camera was connected to a PC; SPLICCO dedicated software was used to record the output signals.

## 3. Testing the Polarization Interferometer

**Figure 3:**
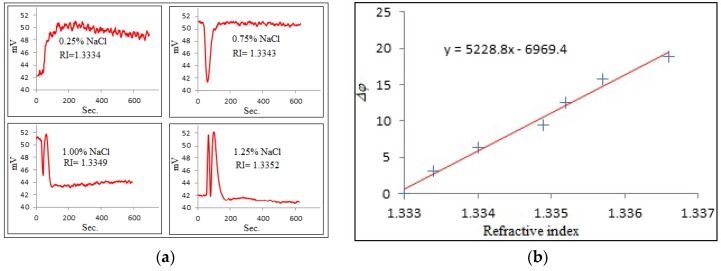
Evaluation of refractive index sensitivity: (**a**) response signals to refractive index changing, (**b**) dependence of phase shift (in rad) against refractive index.

The sensitivity of the waveguide was initially tested by injecting NaCl aqueous solution of different concentrations into the cell. Multiperiodic output signals were recorded, and the number of periods of signal oscillations was roughly estimated from these waveforms. The results of these tests are presented in Figure 3.

The refractive index sensitivity (RIS) of PW sensors can be estimated as a gradient of the above linear dependence:

RIS=2πN/Δn

where *N* is the number of periods of oscillations and $\Delta n=n_{NaCl}-n_{water}$ is the change in the refractive index of liquid medium. The obtained average refractive index sensitivity was around 5200 radians per refractive index unit (RIU), which was more than double compared to the earlier version of the PI experimental setup (Nabok, 2017) and close to the values reported for MZ PW sensors (Nabok, 2016). The achieved sensitivity is much higher than that in other traditional optical methods such as TIRE (total internal reflection ellipsometry) or SPR (surface plasmon resonance).

## 4. Immunosensing Tests on Detection of Ochratoxin A

To prepare the system for detection of mycotoxin molecules, we used electrostatic immobilization of proteins. First, a positively charged layer of poly-allylamine hydrochloride (PAH) was deposited, followed by adsorption of protein A molecules, which are negatively charged, in Tris-HCl buffer, pH 7. Finally, monoclonal antibodies to ochratoxin A (in Tris-HCl buffer) were bound to protein A, and the sensor was ready for detection of ochratoxin A (OTA). All the chemicals used were purchased from Sigma-Aldrich, Dorset, UK.

**Figure 4:**
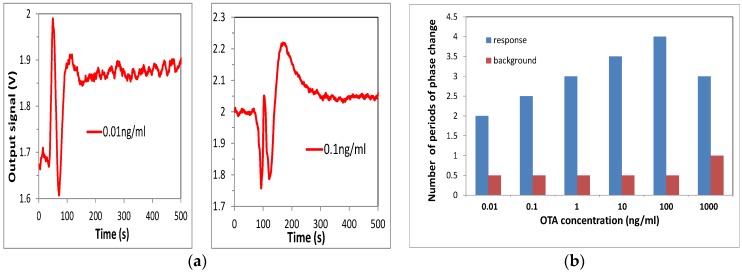
(**a**) Typical sensor responses to binding of 0.01 ng/mL and 0.1 ng/mL of ochratoxin A (OTA) to specific antibodies; (**b**) dependence of PI sensor response on concentration of OTA.

Biosensing tests were performed by injection of OTA solution in water of different concentrations starting from the lowest: 0.01 ng/mL, 0.1 ng/mL, 1 ng/mL, 10 ng/mL, 100 ng/mL, and 1000 ng/mL. The sensor responses were recorded, and the typical responses to 0.01 ng/mL and 0.1 ng/mL of OTA are shown in Figure 4a.

**Figure 5:**
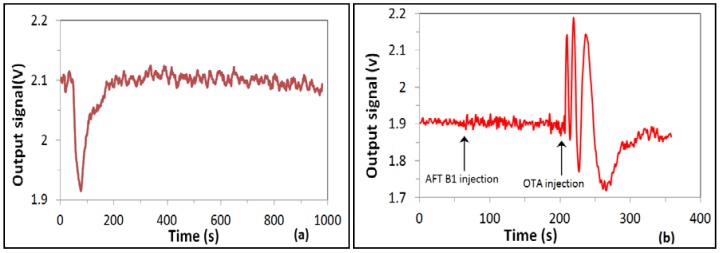
Control tests: (**a**) typical PI sensor response to washing out nonspecifically bound OTA (1 ng/mL), (**b**) typical response to injection of nontargeted analyte, aflatoxin B1 (1 ng/mL); response to injection of 1 ng/mL of OTA is given for comparison. Negative control tests were also carried out by injecting a different toxin (aflatoxin B1), which is not supposed to be bound to anti-OTA antibodies. As shown in (**b**), the response to aflatoxin B1 was comparable to the noise level, indicating high specificity of OTA detection.

The results of these tests are summarized in Figure 4b as the dependence of the phase shift against the concentration of OTA. The sensor response increased in a wide range of concentrations from 0.01 to 100 ng/mL, then decreased at a high concentration of 1 g/mL due to the saturation of bioreceptors. The results obtained are similar to those reported earlier for detection of aflatoxin B1 (Nabok, 2017), though the RIS value and thus the signal clarity were much better. Such biosensing tests were repeated several times; while the waveforms looked slightly different each time because of different initial phase conditions, the total values of a phase shift looked similar, with an accuracy of about 10%. Control test measurements were carried out after each step of OTA binding by purging of about 1 mL of pure Tris-HCl buffer in order to wash out nonspecifically bound OTA molecules. Such tests typically result in a half-period of phase change (see Figure 5a), which corresponds well to observations in MZ-based biosensors [14]. Corresponding background phase changes are also given in Figure 4b. After subtracting the π radians background level, a phase shift corresponding to binding of 0.01 ng/mL of OTA is about one period (or 2π radians). This means that the detection limit could be at least one order of magnitude smaller, thus reaching ppt level or even below, an absolutely remarkable outcome, especially considering the direct immunoassay format used.

## 5. Conclusions and Future Work

The experimental setup of a planar polarization interferometer was developed and tested. After several stages of development, a refractive index sensitivity of 5200 rad/RIU was achieved. A series of biosensing experiments for detecting ochratoxin A in direct immunoassay with specific antibodies were successful; the biosensor was capable of detecting 0.01 ng/mL of ochratoxin A. The work is currently under way. Further development will focus on (i) improving the planar waveguide sensor design using photolithography to make several narrow waveguide channels for simultaneous detection of several mycotoxins, and (ii) developing the data acquisition system using NI card and LabView software. Significant improvements in sensor performance and sensitivity are expected in the near future.
